# Positive Attention Bias Trained during the Rethink Therapeutic Online Game and Related Improvements in Children and Adolescents’ Mental Health

**DOI:** 10.3390/children9111600

**Published:** 2022-10-22

**Authors:** Oana A. David, Silvia Magurean

**Affiliations:** DATA Lab, The International Institute for the Advanced Studies of Psychotherapy and Applied Mental Health, Babeş-Bolyai University, 400015 Cluj-Napoca, Romania

**Keywords:** attentional bias training, serious games, children, adolescents, mental health promotion

## Abstract

Attentional bias towards positive stimuli is considered a resilience factor for mental health and well-being. The aim of the present study was to analyze the effects of an attentional bias training for positive faces in a preventive therapeutic game for children and adolescents. The sample of 54, which consisted of children and adolescents aged between 10–16 years, played the REThink game, which included an attentional bias training level based on the visual search paradigm, where children had the task to quickly find the happy face among other angry faces. We measured mental health, and positive and negative emotions and analyzed their associations between changes in attention bias. Attentional bias indicators demonstrated acceptable reliability and results showed that increases in attentional bias towards positive faces were associated with improvements in children and adolescents’ conduct problems, hyperactivity, and peer relationship problems. Overall, our results support the protective role of training attentional bias towards positive faces as part of a preventive therapeutic game for children and adolescents.

## 1. Introduction

Attentional bias towards threatening stimuli is associated with the development and maintenance of anxiety disorders, as a number of reviews and meta-analysis concluded (see [1,2,3]). Based on this, researchers developed implicit methods to reduce or re-train attentional bias, which can have rather small, but reliable effects for anxiety (see [4] for a meta-analysis). Indeed, computer-based experimental paradigms have been developed that can reduce the excessive attention allocation to anxiety-relevant information (e.g., negative cognitive processing, cognitive distortions [5,6,7,8]). The visual search task is such a probe that asks the participants to quickly and repeatedly find a target disorder-incompatible stimulus, such as a smiling face, among disorder-relevant stimulus, such as angry faces, so that they implicitly learn how to develop a pattern of selective attention in favor of positive information.

Several studies also investigated the protective role that attentional bias towards positive stimuli might have for mental health (e.g., [9,10]). For instance, Thoern and colleagues [11] showed that people with stronger bias towards positive faces report higher levels of trait resilience. Moreover, using an experimental perspective, Dandeneau and colleagues [12] demonstrated that training attentional bias towards positive faces increased self-esteem, decreased cortisol levels and perceived stress responses, and improved performance and self-confidence. Therefore, attentional bias towards positive stimuli seems to have a protective role for mental health.

The effects of positive biases were analyzed in children and adolescents as well, although to a lesser degree. There is evidence showing that training anxious children to disentangle attention from threatening stimuli reduced stress vulnerability and anxiety [13]. Additionally, training anxious children to find happy faces among angry faces decreased diagnosis severity [14,15].

Considering the protective role of the attentional bias towards positive emotions, and previous effects found with children and adolescents, it is important to incorporate attentional bias training for positive stimuli (ABT) into evidence-based prevention programs for children and adolescents. Furthermore, since the ABT is a computerized test, this can be easily incorporated into therapeutic games (serious games), which are a new approach to increase the accessibility of mental health programs (for a review, see [16,17]). 

To our knowledge, previous attempts to integrate attentional bias training in an evidence-based serious game approach are scarce (i.e., *Shots* for alcohol attentional bias [18]; *PsychMeUp* [19]). In this context, we designed an attentional bias training towards positive faces game level and integrated it into a complex serious game for children and adolescents. The *RET*hink game was designed to increase resilience through acquiring healthy strategies for coping with negative emotions such as anxiety, anger, and depression. *RET*hink focuses on developing rational thinking by replacing irrational beliefs with their alternative rational believes, in a Rational Emotive Behavioral Therapy framework (REBT [20]). Efficacy of the *RET*hink game was investigated in a clinical trial (ClinicalTrials.gov Identifier: NCT03308981) and was found to have a preventive effect in healthy children and adolescents by reducing emotional symptoms and depressive mood, while increasing their emotional regulation ability [21].

An ABT for positive stimuli (happy faces) was integrated as part of the final *RET*hink level. The current study is a secondary data analysis of the study regarding the effectiveness of the *RET*hink game [21]. The aim of the present study was to investigate the efficacy of the ABT skills level as part of *RET*hink in training attention towards positive faces and their association with psychological changes in emotional symptoms. We proposed that changes in attentional bias would be associated with the improvements in children and adolescents’ emotional and behavioral symptoms, thus supporting the beneficial role of the attentional bias training as part of the *RET*hink game. We thus expected that increases in attentional bias towards positive stimuli to be associated with improvements regarding children’s psychological functioning (emotional and behavioral symptoms) and their mood (e.g., negative emotions, positive emotions).

## 2. Methods

### 2.1. Participants 

The sample (*N* = 54) consisted of children and adolescents, aged between 10–16 years, who volunteered to participate in the clinical trial presented in David et al. [21,22] and were assigned to the *RET*hink condition. Six participants did not complete the initial assessment and therefore the final sample of participants who completed the program consisted of 48 children (36 girls and 12 boys) with a mean age of 13 years (SD = 2.06). Twenty-two of them were aged 9–12 and 26 were aged 13–16. There was no inclusion or exclusion criteria used in this study. Data on attentional bias from six participants were lost due to technical issues. Out of the remaining 42 participants with valid attentional bias data, eight participants did not complete the intermediary assessment. Therefore, a final sample of 34 participants was included in the main analysis of associations between changes in attentional bias and changes in emotional symptoms.

### 2.2. Procedure

All experimental protocols were approved by The Babes-Bolyai University Ethics Committee (Approval Number: 30970/10-12-2016) and informed consent was obtained from the parents/their legal guardian(s) and the school principal. The study protocol was registered at ClinicalTrials.gov: NCT03308981. All methods were carried out in accordance with relevant guidelines and regulations. The sample size was estimated for the main trial to test the efficacy of the *RET*hink intervention so that we can detect at least a medium-to-large effect size (Cohen’s d = 0.60). With set parameters for type I error probability of α = 0.05, in a two-tailed test, and a statistical power of at least 0.80, we computed a needed sample size of 45 participants per group. In order to investigate whether attentional bias indicators are associated with changes in mood and emotional and behavioral symptoms, we computed delta change scores as a proportion of the difference between the two assessments. In the case of attentional bias, the delta change indicator reflected the proportion of change from the first session to the second session. Therefore, higher attentional bias delta change represented increased bias towards positive stimuli from the first to the second session (since the reaction time indicator decreased). For the questionnaires, the delta change indicator was computed as the proportion of change from the intermediary assessment to the post-intervention assessment, since the attentional bias training was performed between these two moments (during modules six and seven). Higher delta change indicators for the questionnaires represented decreases in scores from intermediary to post-intervention assessment. We computed within-subject adjusted Cohen’s d index of effect size, based on recorded means and standard deviations on the two points time on attention bias.

#### 2.2.1. *RET*hink Game 

**Figure 1:**
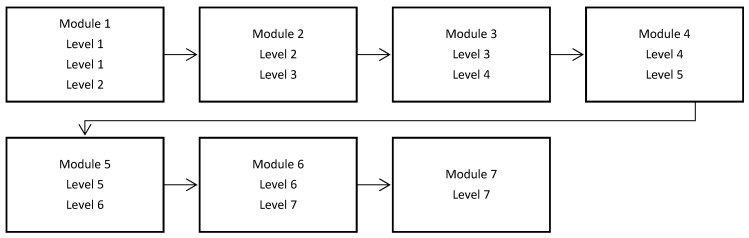
*RET*hink game modules and the corresponding levels.

*RET*hink is a therapeutic online game designed as an iOS application for building resilience in children and adolescents and promoting mental health. The game has a main positive character, RETMAN, whose mission is to help people on Earth against *Irrationalizer*, who is a “bad character” because of his powers to create unhealthy emotions and inoculate irrational thinking. The game comprises seven levels addressing specific therapeutic objectives: Level 1: identifying the emotional reactions, differentiating between basic emotions, complex emotions, and functional and dysfunctional emotions; Level 2: identifying cognitive processes; Level 3: identifying the relation between cognitive processes, emotions, and behavioral reactions, Level 4: changing irrational cognitions into rational cognitions; Level 5: building problem solving skills; Level 6: building relaxation skills; and Level 7: consolidation of previous skills and building happiness skills. The attentional bias training was included as part of Level 7. For a detailed description of the game, see the studies regarding the development and efficacy of the *RET*hink therapeutic game [21,23] and, respectively, its mechanisms of change [22]. Main outcomes of the trial documented to be improved by playing the *RET*hink game were emotional problems and emotional regulation abilities, secondary outcomes were temperament and emotional distress, and documented mechanisms were irrational beliefs. Participants played each level twice, organized in seven gaming modules, in order to consolidate the skills cultivated by each level (see Figure 1). Going through one module required approximately 50 min. Children and adolescents played the game on an Apple iPad Air 2 during one month. 

Three assessment moments were included: a pre-intervention assessment, an intermediary assessment after module 4, and a post-intervention assessment after all the levels were finished. Because the ABT was performed between the intermediary and the post-intervention assessment, we used only these two assessment moments to analyze change related to ABT.

#### 2.2.2. Attentional Bias Training

**Figure 2:**
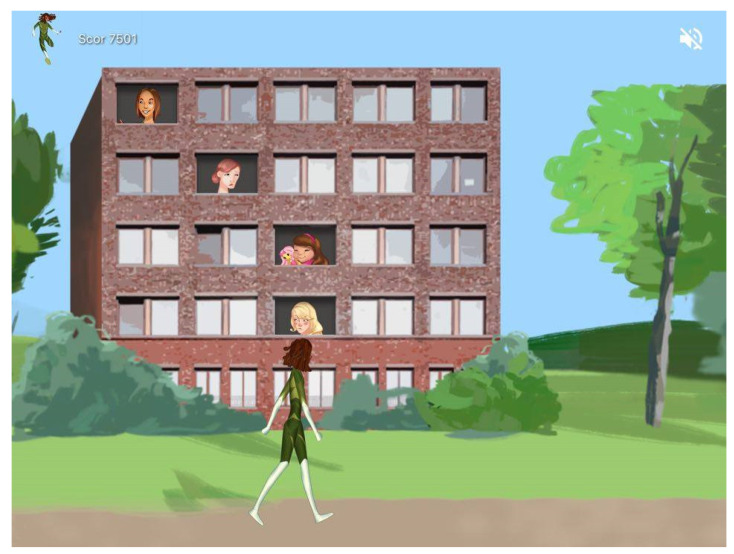
Illustration of the attentional bias training task.

The ABT was part of Level 7 (sub-level 7.1) which was designed to consolidate previous skills and develop happiness skills. The attentional bias training used a visual search paradigm comparable to the intervention presented by Dandeneau and colleagues [12]. For the attentional bias training, participants were presented with the image of an apartment building with windows (see Figure 2). The player had the mission to watch the faces which appeared in the windows and find the happy face among the angry faces as fast as possible. The task was thus to quickly identify the happy face and touch it on the iPad screen. Reaction time was registered for each trial from the moment the faces appeared on the screen until the participant touched one of the faces. 

The difficulty of the task increased across eight blocks, comprising three trials each, as the number of total faces which appeared simultaneously increased from 9 to 30. For each block, the first trial was considered as a practice trial, so that only reaction times from the second and third trials of each block were included in the analysis. When an error was registered for a trial (i.e., participant identified an angry face instead of the happy one) participants automatically restarted the respective block, until each block was completed correctly. Only blocks without errors were included in the analysis.

As with the rest of the game, participants went through the positive ABT twice, once during the sixth module, and again during the seventh module. Participants’ reaction times during the two sessions of attentional training were used as an attentional bias indicator. For each of the training sessions, we computed the mean reaction time of all second and third trials in the eight blocks, as an indicator for attentional bias. To test the reliability of the positive ABT gaming procedure, we performed a split-half analysis. We correlated the mean reaction time of second trials from all blocks with the mean reaction time of third trials from all blocks. First trials were considered practice trials and were not included in any of the analysis. The split-half reliability for the first attentional bias session (in module 6) was 0.55, and 0.39 for the second session (in module 7). Even though reliability of the attentional bias is rather low, especially for the second session, it is comparable to previous findings [24].

### 2.3. Measures

*Strength and Difficulties Questionnaire* (*SDQ;* [25,26]) is a 25-item self-report behavioral screening. The scale measures prosocial behavior as a strength, and four types of difficulties: emotional symptoms, conduct problems, hyperactivity/inattention, and peer relationship problems. Items are rated on a 3-point Likert scale ranging from 0 (“not true”) to 3 (“certainly true”). The scores for each subscale is obtained by summing the scores for all items (e.g., I think before I do things) comprising the subscale. Therefore, higher scores represent higher levels of difficulties and, respectively, higher levels of prosocial behavior. Internal consistencies found in the main study for SDQ are α = 0.75 for emotional symptoms subscale, α = 0.80 for the total level of psychological difficulties, α = 0.65 for conduct problems subscale, α = 0.65 for hyperactivity-attention subscale, α = 0.63 for peer problems subscale, and α = 0.67 for prosocial behavior subscale [27]. 

*Functional and Dysfunctional Child Mood Scales—girls and boys versions* (FD-CMS; [28]) is a 9-item self-report scale based on the binary model of distress [29]. Each item represents an emotion using images related to the specific emotion (e.g., sadness). Participants use a 10-point Likert scale ranging from 0 (“no emotion”) to 10 (“strong emotion”) to appraise the intensity of the emotions during the last week. The items represent functional negative emotions (sadness, anxiety, and irritation), dysfunctional negative emotions (depression, fear, and anger), and positive emotions (happiness, trust, and silence). The scores for each dimension is generated by summing all scores for all items included in the dimension, so that higher scores are indicative of higher intensity of the corresponding emotions during the past week. The measure registered adequate reliability in a preliminary study [28] and internal consistencies obtained for FD-CMS in our sample are α = 0.80 for functional negative emotions subscale, α = 0.65 for dysfunctional negative emotions subscale, and α = 0.66 for positive emotions subscale.

## 3. Results

### 3.1. Attentional Bias

The attentional bias indicator (the mean of all valid trials) of the first training session was positively correlated with the attentional bias indicator of the second training session *r*(40) = 0.49, *p* < 0.01. The difference between the first and the second section is not statistically significant *t*(41) = 1.09, *p* = 0.27, with a slightly higher reaction time for the first session as compared to the second session (see Table 1, Cohen’s *d* = 0.17).

### 3.2. Changes in Attentional Bias and Changes in Psychological Functioning

We proposed that changes in attentional bias would be associated with the improvements in children and adolescents’ emotional and behavioral symptoms, thus supporting the beneficial role of the attentional bias training as part of the *RET*hink game.

In order to investigate whether attentional bias indicators are associated with changes in mood and emotional and behavioral symptoms, we computed delta change scores as a proportion of the difference between the two assessments. In the case of attentional bias, the delta change indicator reflected the proportion of change from the first session to the second session. Therefore, higher attentional bias delta change represented increased bias towards positive stimuli from the first to the second session (since the reaction time indicator decreased). For the questionnaires, the delta change indicator was computed as the proportion of change from the intermediary assessment to the post-intervention assessment, since the attentional bias training was performed between these two moments (during modules six and seven). Higher delta change indicators for the questionnaires represented decreases in scores from intermediary to post-intervention assessment.

The results are showing that children and adolescents who performed better in the second attentional bias training session also registered reductions in conduct problems (*r*(32) = 0.48, *p* < 0.01, one-tailed), hyperactivity (*r*(32) = 0.33, *p* = 0.02, one-tailed), and peer relationship problems (*r*(32) = 0.36, *p* = 0.01, one-tailed) from the intermediary assessment to the post-intervention assessment. 

Increases in attentional bias towards positive stimuli seemed to also be associated with increased intensity of the dysfunctional negative emotions, though the effect size is rather small (*r*(32) = 0.28, *p* = 0.07, two-tailed). Changes in attentional bias were not associated with any changes in positive or functional negative emotions (see Table 1). 

## 4. Discussion

In this study, we aimed to investigate if an attentional bias training for positive faces integrated in a therapeutic game is associated with changes in psychological and emotional functioning.

The reliability of the attentional bias indicator was rather low, although comparable to reliability of attentional bias in previous studies [24]. Attentional bias during the first training session was positively correlated to the attentional bias during the second session. Mean reaction time from the first session did not differ significantly from the mean reaction time of the second session of attentional bias, even though the performance improved with a small effect size (lower reaction times) during the second session. 

Further on, we analyzed how changes in attentional bias are associated with changes in emotional symptoms. Our results suggest that attentional bias training had a positive effect on children and adolescents’ psychological functioning. Participants who improved their attentional bias towards positive stimuli also reported reductions in conduct problems, hyperactivity, and peer relationship problems from the intermediary assessment to the post-intervention assessment. These results are in line with previous findings regarding the positive effect of attentional bias training on children’s and adolescents’ mental health [15], and support the use of attentional bias training as a component add-on in preventive programs for children and adolescents. We did not find, however, such significant associations of positive attentional bias with the improvements in emotional symptoms (main outcome) or prosocial behaviors. This might be explained by our results documenting irrational beliefs as the mechanisms of change for the improvements regarding emotional symptoms for children playing the *RET*hink game [22]. Therefore, implicit positive attentional bias trained in the *RET*hink therapeutic game based on a gamified visual search task seems to be associated with children and adolescents’ behavioral improvements.

Surprising results were observed for the association between changes in attentional bias and changes in participants’ positive mood. We expected a positive relation between attentional bias towards positive stimuli and positive emotions, which was not supported in our study. This result needs to be seen in the light of the results of other studies investigating the effect of gamified versions of attention training, which documented changes in attention bias but failed to document effects on the youth’s mood [30] or behaviors [18]. Moreover, attentional bias training sessions might be needed to lead to an observable effect on positive emotions intensity, since a review by Hakamata et al. [31] noticed that the number of sessions has a significant effect on attentional bias. 

A small unanticipated effect was found for the association between increases in positive attentional bias and increases in negative dysfunctional emotions. Children and adolescents with improved attentional bias scores (from the first to the second session) experienced slightly lower improvements in their negative dysfunctional emotions. This might be in line with our previous explanation of the low associations with emotional symptoms, and might suggest that training youths to observe positive faces around them does not imply that they would experience less negative emotions and thus different mechanisms might be involved in this path (i.e., reducing their irrational thinking as found by our mechanisms of change analysis [22]).

## 5. Limitations and Future Research

Our study has some limitations which need to be addressed. In the absence of a control group to play the game without the attentional bias training it is difficult to disentangle the effects of the other modules from the effect of the attentional bias training. Thus, future studies will need to measure outcome before and after the attention bias training level alone, in order to depict its isolated effects. Moreover, our positive attentional bias indicator was recorded during the attentional bias training, and not as a separate assessment task, which was also reflected in a rather low reliability of the attentional bias indicator. As a previous review revealed that the number of sessions has an effect on attentional bias [31], more sessions could yield stronger effects and could have helped clarify some unanticipated relationships we observed in our study. Indeed, since the attentional bias towards positive emotions seem to have a protective role in children and adolescents, it can be advisable to incorporate attentional bias training for positive stimuli into evidence-based prevention programs for children and adolescents. Additionally, offering training to parents of children at risk of anxiety disorders in positive bias modification might bring long-term benefits for the intergenerational transmission of the negative attention bias patterns [32,33,34,35,36]. Future studies need to measure the mechanisms of change regarding negative cognitive processing, using reliable implicit and explicit measures [37,38,39,40], during an anxiety-inducing task or in ecological situations. Also, more proximal mental health resilience outcomes need to be measured together with biological reactivity to threat in relation to attention bias training.

In sum, prevention programs for children and adolescents designed in the form of therapeutic games, such as the *RET*hink online game, can easily incorporate attentional bias training components for positive stimuli. The online gamified version of attention bias training offers an accessible and promising tool that can have positive effects on the behavioral and social functioning of the youths. Overall, our results are in support of the use of attentional bias training for positive stimuli in children and adolescents as a preventive measure to be integrated in more complex therapeutic preventive programs for extending their efficacy. 

## Figures and Tables

**Table 1:** Correlations between changes in attentional bias and changes in mental health outcomes.

		1	2	3	4	5	6	7	8	9	M1(SD)	M2(SD)
1	AB	--									4.13 (0.96)	3.96 (1.00)
2	SDQ-ES	0.14	--									
3	SDQ-CP	0.48 **	0.49 **	--								
4	SDQ-H	0.33 *	0.41 **	0.51 **	--							
5	SDQ-PR	0.36 *	0.40 **	0.37 *	0.31 *	--						
6	SDQ-PS	0.09	−0.07	−0.23	−0.14	−0.23	--					
7	FD-CMS-F	−0.09	0.49 **	−0.18	0.04	0.15	0.29 *	--				
8	FD-CMS-DF	−0.28 *	0.16	−0.24	−0.08	−0.04	0.26	0.40 **	--			
9	FD-CMS-P	−0.02	−0.26	−0.17	−0.13	−0.18	0.50 **	0.05	0.05	--		

* *p* < 0.05, ** *p* < 0.01, one-tail. AB = attentional bias; SDQ-ES = SDQ emotional symptoms; SDQ-CP= SDQ conduct problem; SDQ-H = SDQ hyperactivity; SDQ-PR= SDQ peer relationship problems; SDQ-PS = SDQ prosocial behavior; FD-CMS-F = FD-CMS functional negative emotions; FD-CMS-DF= FD-CMS dysfunctional negative emotions; FD-CMS-P= FD-CMS positive emotions.

## Data Availability

Datasets used and/or analyzed during the current study is available from the corresponding author on reasonable request.
